# Diminished Returns of Parental Education in Terms of Youth School Performance: Ruling out Regression toward the Mean

**DOI:** 10.3390/children7070074

**Published:** 2020-07-07

**Authors:** Shervin Assari, Shanika Boyce, Mohsen Bazargan, Cleopatra H. Caldwell

**Affiliations:** 1 Department of Family Medicine, Charles R. Drew University of Medicine and Science, Los Angeles, CA 90059, USA; Mohsenbazargan@cdrewu.edu; 2 Department of Pediatrics, Charles R. Drew University of Medicine and Science, Los Angeles, CA 90059, USA; ShanikaBoyce@cdrewu.edu; 3 Department of Family Medicine, UCLA Health, Los Angeles, CA 90095, USA; 4 Department of Health Behavior and Health Education, University of Michigan, Ann Arbor, MI 48109, USA; cleoc@umich.edu

**Keywords:** race, ethnicity, educational attainment, African Americans, Blacks, socioeconomic status, school performance, regression toward the mean

## Abstract

Background: Minorities’ Diminished Returns (MDRs) refer to systemically weaker effects of socioeconomic status (SES) indicators on various developmental, behavioral, and health outcomes of ethnic minorities compared to non-Hispanic (non-Latino) Whites. Similar MDRs also exist for the effects of parental education on the school performance of ethnic minority youth. Aim: To assess whether regression toward the mean (RTM) has any role in explaining the diminished effects of parental education on the school performance of Black and Hispanic youth relative to non-Hispanic White youth. Materials and methods: Data for this cross-sectional study came from the Monitoring the Future survey (MTF, 2017), a nationally representative survey of American youth in 12th grade. The sample included 10,262 youth who were 12th graders (typically 17–18 years old). The independent variable was parental education with five categories: Some high school, High school graduate, Some college, College graduate, and Graduate school. The outcome was self-reported school performance measured as grade point average (GPA). Ethnicity was the effect modifier. Analysis of variance (ANOVA) and the Tukey Post Hoc test was used to analyze the data. Data visualization (line graphs) was used to visualize the shape of youth GPA as a function of parental education levels across ethnic groups. Results: While a perfect stepwise increase was seen in youth school performance as a result of parental education improvement, this pattern differed considerably across ethnic groups. Such a perfect stepwise increase in youth school performance as a result of the incremental increase in parental education was missing for Black and Hispanic youth. The shape of the association between parental education and youth school performance ruled out regression toward the mean (RTM) as an explanation for the observed diminished effects of parental education on the school performance of Black and Hispanic youth. Conclusion: Diminished returns of parental education on the school performance of Black and Hispanic youth cannot be explained by regression toward the mean. Other factors and contextual processes, such as segregation, discrimination, racism, and poor quality of schools in urban areas, should be investigated in future research.

## 1. Introduction

“Minorities’ Diminished Returns” (MDRs) refers to the smaller benefit of socioeconomic status (SES) resources for Blacks and Hispanics than for non-Hispanic Whites [1,2]. The literature on MDRs suggests that educational achievement, whether one’s own [3] or that of one’s parents [4], is associated with unequal outcomes across diverse ethnic groups. Compared to non-Hispanic (non-Latino) Whites, Hispanics and Non-Hispanic Blacks experience fewer benefits from their own and their parents’ education, across a wide range of tangible outcomes [2,5,6].

In line with the MDRs phenomenon, educational achievement differentially translates to tangible outcomes for non-Hispanic White families and Black families [1,2]. Among adults, educational attainment shows a weaker negative association with smoking [7], drinking [8], poor diet [9], obesity [10], depression [11], suicidality [12], and mortality [13] for Blacks and Hispanics than for non-Hispanic Whites. Researchers have replicated these findings for children [14], youth [15], adults [11], and older adults [16,17].

MDRs are frequently exhibited in youth. MDRs of family SES on youth outcomes is a mechanism by which ethnic disparities are transmitted from one generation to the next [6,10,18]. Many studies [6,10,18] have shown weaker effects of family SES on youth body mass index (BMI) [10], self-rated health (SRH) [18], attention deficit hyperactivity disorder (ADHD) [19], mental health [20], and impulse control [6] for Black and Hispanic youth than non-Hispanic White youth. Similarly, parental education also shows a weaker association with upward educational mobility [4], school bonding [21], and school performance [22] for Black and Hispanic youth than non-Hispanic White youth.

Researchers have explored several potential underlying mechanisms to explain the MDRs phenomenon. One potential explanation is the close proximity of high SES Hispanic and Black families to non-Hispanic White families, which means an increase in discriminatory experiences [23]. Extensive research has shown a link between discrimination and poor outcomes across domains [24,25]. Another explanation for MDRs may be the low quality of education in urban areas where most ethnic minorities attend schools [26,27]. Finally, highly educated ethnic minorities are more likely to work in worse jobs, which are associated with more stress and less income [28]. As a result, highly educated ethnic minorities accumulate less wealth than non-Hispanic Whites. Thus, MDRs of education may be due to labor market discrimination.

Why MDRs exist is still unclear. Some scholars have attributed the MDRs to the lower quality of education in urban areas where most Black and Hispanic children and youth attend schools. Structural and institutional racism impact the practices and preferences of the labor market [5]. As society differently treats sub-populations, Blacks and non-Hispanic Whites differ in how well they can mobilize their education to secure desired outcomes [1,2]. Non-Whites must expend higher levels of psychosocial effort than Whites in order to achieve the same upward social mobility [4]. Non-Whites consistently put additional psychological and physiological effort into climbing the social ladder, in comparison to Whites [4]. Given the history and legacy of slavery and Jim Crow over centuries and even what is left of that legacy today (e.g., segregation and discrimination), education produces better gains in income, social power, and purchasing power for Whites than for non-Whites [1,2]. As a result, similar educational credentials have a greater positive impact on the living conditions of non-Hispanic Whites than on that of Blacks [1,2]. Blacks are more likely to gain their education in an inner-city school, which are low in resources. Black children and youth commonly experience discrimination [29,30]. Such discrimination increases the risk of problems across domains [24,25,31]. Discrimination may result in MDRs of education [32,33]. These processes are likely to reduce the impact of one’s own and one’s parents’ education on tangible outcomes for Black and Hispanic individuals compared to non-Hispanic White people. Some critics of MDRs, however, have proposed that Regression Toward the Mean (RTM) may explain MDRs. No previous studies, however, have ever explored whether RTM can explain MDRs.

In brief, RTM can be characterized by the high likelihood of values close to the average (mean) of the dependent value for extreme levels of an independent variable [34,35,36]. RTM frequently leads to inaccurate conclusions, wrong inferences, and spurious associations. RTM is observed both in cross-sectional and longitudinal data; however, it is more commonly discussed for repeated measures in the presence or absence of an intervention or a treatment [34,35,36]. Given that RTM has the potential to lead to wrong conclusions, it has been examined extensively in the literature [37,38,39]. This literature shows that RTM is a common problem and source of bias in the health sciences [26,27,37,38,39,40,41]. However, the investigator is not aware of any previous studies that have explored the role of RTM as an explanation for observed MDRs. In other words, it is unknown if the pattern of associations in the MDRs theory follows the expectations that occur in RTM. This is of increasing importance, given that MDRs are receiving growing attention [3,16,19,21,42,43,44,45,46] and have significant policy implications [1,47].

This study aimed to investigate the role of RTM in explaining MDRs of parental education on school performance (academic achievement) of ethnic minority youth. The hypothesis was that RTM does not explain the MDRs of parental education on school performance in ethnic minority groups. To produce generalizable results, a nationally representative sample of non-Hispanic White, Black, and Hispanic youth was used.

## 2. Materials and Methods

### 2.1. Design and Settings

This cross-sectional study is a secondary analysis of existing data from the Monitoring the Future (MTF-12th grade) survey. The MTF is a nationally representative survey of American 12th-grade students. The primary aim of the study is to examine the epidemiology and risk factors of youth substance use in the United States. The MTF-12th grade enrolls a national sample of 12th graders that are being followed into adulthood. In the United States, almost 3.8 million students are enrolled in the 12th grade [48]. The MTF used variables such as gender, ethnicity, and urbanity to generate a nationally representative sample.

### 2.2. Sample and Sampling

The 2017 MTF-12th grade study enrolled individuals who were enrolled in their 12th grade. Exclusion criteria: Not reporting school performance, not reporting ethnicity, not reporting education for either parent, not having any parent in the household, not reporting the number of parents in the household, not being in 12th grade, and Hispanic ethnicity (measured as self-identified). The analytical sample in this study was 10,262 youth.

### 2.3. Study Variables

*Ethnicity.* Ethnicity was measured as self-identified. Ethnicity in the current study was a nominal variable, and the moderator variable (non-Hispanic White, Hispanic, and Black).

*Parental education.* Parental education was a five-level variable as below: 1 = “Some high school,” 2 = “Completed high school,” 3 = “Some college,” 4 = “Completed college,” 5 = “Graduate or professional school after college.” This variable was a nominal variable.

*School Performance.* Participants’ school performance was measured by asking participants to report their grade point average (GPA). The exact item was, “What is your current overall school performance?” The possible answers were 9 = A (“93–100”), 8 = A- (“90–92”), 7 = B+ (“87–89”), 6 = B (“83–86”), 5 = B- (“80–82”), 4 = C+ (“77–79”), 3 = C (“73–76”), 2 = C- (“70–72”), and 1 = D (”69 or below”). School performance was a continuous measure, with a potential range from 1 to 9. A higher score indicated better school performance.

### 2.4. Statistics

The investigator used SPSS 23.0 (IBM Inc., Armonk, NY, USA) for data analyses. School performance, a continuous variable, was the outcome. Parental education, a 5-level categorical variable, was the independent variable. Ethnicity was the moderator. Analysis of variance (ANOVA) was used to analyze the data. ANOVA was followed using the Tukey Post Hoc test. Line graphs were used for data visualization. We graphed mean school performance (GPA) by parental education and by ethnicity. In our visualization, RTM would be a potential explanation for MDRs, only if the data showed less than expected effects of the SES resource (independent variable = parental education) at the right tail of the distribution (where parental education is highest). That is, RTM would be a possible explanation if and only if diminished returns were most pronounced at the highest level of parental education. However, if diminished returns are consistent throughout all levels of parental education, then RTM can be ruled out as an explanation for MDRs. In other words, if MDRs are seen for any incremental increase in parental education, then RTM is ruled out as an explanation.

### 2.5. Ethics

The MTF study protocol is approved by the University of Michigan Institutional Review Board (IRB). All participants gave a written consent/assent, depending on their age at the time of the survey. For the participants who were younger than 18 years old, their parents also signed informed consent.

## 3. Results

### 3.1. Univariate Analysis

The sample was composed of 10,262 youth who were all 12th graders. This sample was composed of Hispanics (22.75%), non-Hispanic Blacks (14.90%), or non-Hispanic Whites (62.35%). The sample contained slightly more females (51.6%) than males (48.4%) (Table 1).

Table 2 also described the educational attainment across ethnic groups. As this table shows, non-Hispanic Whites had the highest education, followed by non-Hispanic Blacks. Hispanics had the lowest level of education.

### 3.2. ANOVA Results

Table 3 shows the results of four sets of ANOVAs, one in the pooled sample, then one in non-Hispanic Whites, one in non-Hispanic Blacks, and one in Hispanics. Then, Table 4 and Table 5 show the results of Tukey Post Hoc test to better understand the ANOVAs presented in Table 3. While a stepwise increase in youth school performance was present as parental education improved in the pooled sample, this pattern differed across various ethnic groups. The stepwise increase in school performance was perfect only for the pooled sample, meaning that with no exception, an increase in parental education was always associated with an increase in school performance. For non-Hispanic White youth, this pattern was almost perfect. However, for non-Hispanic Black and Hispanic youth, the effects of an incremental increase in the level of parental education did not always enhance the student’s school performance, as this pattern was irregular. Thus, there was no stepwise increase in school performance as a result of parental education for ethnic minority youth.

### 3.3. Data Visualization

**Figure 1:**
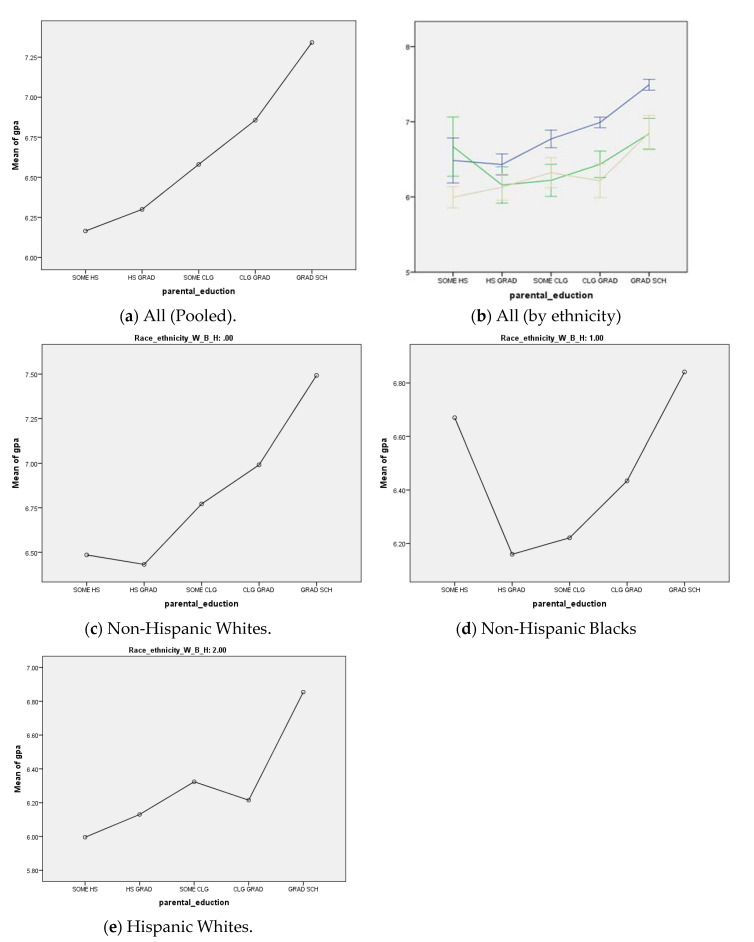
Association between parental education and school performance in Non-Hispanic Whites (NHWs), Non-Hispanic Blacks (NHBs), and Hispanic Whites (HWs). (**a**) All (Pooled); (**b**) All (by ethnicity); (**c**) Non-Hispanic Whites; (**d**) non-Hispanic Blacks; (**e**) Hispanic Whites.

We drew five line-graphs. These graphs present mean youth school performance based on parental education. The first two graphs are for the overall sample, first pooled, and then separate lines for each ethnic group. Then, we added three similar graphs; one for Blacks, one for Hispanics, and one for non-Hispanic Whites. In the first graph, there was a perfect stepwise increase in GPA as parental education improved in the pooled sample. As the second graph shows, however, this stepwise increase in GPA could be seen for non-Hispanic White youth but not Black or Hispanic youth. The last graphs also showed the most significant deviation for the form of shape for Black youth. For Black youth, a major anomaly could be seen, which was not due to the end tale of the distribution (highest education) but the lowest educational level. As such, our data visualization ruled out the RTM as an explanation for the observed diminished effects (MDRs) of parental education on the school performance of Black and Hispanic relative to White and non-Hispanic youth (Figure 1).

## 4. Discussion

This study documented a stepwise increase in school performance for the overall sample of youth. However, such a stepwise increase in school performance as a result of an increase in parental education was absent for non-Hispanic Black and Hispanic youth. As our data visualization showed, RTM seems not to be a plausible explanation for the observed diminished effects of parental education on the school performance of non-Hispanic Black and Hispanic youth relative to non-Hispanic White youth.

While each additional level of parental education always resulted in a significant enhancement of the 12th graders’ school performance in the US, this effect was diminished for ethnic minorities. In addition, MDRs could be seen at all levels of parental education, not merely at the extreme high of parental education, which would be expected if RTM was the reason behind the MDRs. That is, the effect of parental education on youth school performance was always smaller at all thresholds for ethnic minorities, when compared to Whites and non-Hispanics. Thus, RTM is ruled out as the statistical explanation for MDRs.

A large body of research has documented MDRs of various SES indicators for Hispanics and Blacks compared to Whites and non-Hispanics [3,5,46,49]. Similar MDRs are shown across age groups, SES resources, and outcomes [1,2]. Education results in more gain for White and non-Hispanic than for Black and Hispanic children [14], youth [6,10,18], adults [5], and older adults [8]. We have documented similar MDRs in Blacks [17,22], Asian Americans [43], Native Americans [50], Hispanics [3,51], and members of the LGBTQ community [46].

For youth, MDRs of parental education results in the trans-generational transmission of ethnic inequalities [6,10,18]. Many studies [6,10,18] have shown weaker effects of family SES on youth upward educational mobility [4], school bonding [21], and school performance [22] for Black and Hispanic youth than for non-Hispanic White youth. MDRs are not limited to educational outcomes and extend to health outcomes such as tobacco use [7,52,53], alcohol use [3], body mass index (BMI) [10], self-rated health (SRH) [18], attention deficit hyperactivity disorder (ADHD) [19], mental health [20], and impulse control [6].

As shown by Bumpus, Umeh, and Harris [54], Black youth receive smaller benefits from their parents’ social class than non-Hispanic Whites. They found that for youth not in married households, Blacks gain less benefits from their mothers’ occupational prestige on their youth outcomes (particularly college enrollment) than non-Hispanic Whites [54]. Previous research has shown that these patterns hold for education [3], employment [55], income [19,49,51,56,57], and marital status [58].

The difference in the shape of the association between parental education and school performance across ethnic groups is not limited to the right tail of the distribution and can be seen across education levels. This pattern rules out RTM as an explanation for MDRs. We have previously argued that MDRs may be due to racism and discrimination in the US education system and labor market. As a result of racism, segregation, and social stratification, schools have fewer resources in the areas where ethnic minorities receive an education [29,59]. Another mechanism of the MDRs is the US labor market. Due to differential treatment, racism, segregation, and discrimination by the US labor market, Black Americans work in worse jobs than White Americans at all levels of education [60]. Thus, at higher levels of education, Black and Hispanic parents make considerably less income than White and non-Hispanic parents.

The US social system has increased the psychosocial cost of upward social mobility for Black and Hispanic families. Being charged with extra costs for their upward social mobility, Black families gain less from their education. Upward social mobility is qualitatively different for ethnic groups, being more difficult for Blacks than non-Hispanic whites [1,2]. Historically, Blacks have had less political power. Thus, their voice is underrepresented in writing laws and policies. As a result, policies, written by the dominant group, have historically maximized their gain, ignoring the structural barriers and challenges that Blacks and other minority populations deal with daily [1,2].

At each level of family SES, Hispanic and Black parents face disproportionately higher levels of environmental and societal problems in their daily lives. Hispanic and Black families with high SES still experience high levels of discrimination that reduces their outcomes [30,61,62]. In a society that is aware of ethnicity and color, people are often treated based on their skin color and ethnicity rather than their potential. As a result of such prejudice, highly educated Hispanic and Black families do not have the same opportunities and access that their White counterparts do.

Due to existing MDRs [1,2], equal SES resources result in unequal outcomes. Due to the MDRs, ethnic minority groups are at a relative disadvantage compared to the majority group. MDRs conceptualize ethnicity as a social rather than a biological construct [63]. Thus, MDRs-related differences in school performance are due to social processes such as segregation and stratification, as opposed to genetics or IQ [64,65,66]. Ethnicity in the US is a proxy of living conditions, history, and inequality in daily life, as well as racism, discrimination, a legacy of slavery, oppression, and Jim Crow policies [63,67,68,69]. While genes and IQ may also play a role, our focus is on sociological mechanisms [63,67,68,69]. We argue that contextual factors such as neighborhood stress and concentrated poverty may reduce the academic success of high SES youth [64,65,66,70].

Some solutions are increasing political participation of power, social justice, reduction of segregation, and affirmative action policies, as well as reducing any form of discrimination across institutions. Such efforts should be multi-level and include schools, correctional settings, and policing. A decline in stop and frisk, mass incarceration, and affirmative action may be needed.

There is a need for future research. Religious aspects, involvement in sports, parenting, and availability of resources are linked to ethnicity and youth behavior. Future research should test if MDRs of SES in Black families may be related to any of the above factors. Differential engagement in sports, for example, may explain why high SES Black, Hispanic, and non-Hispanic White youth have different GPA school averages. 

## 5. Limitations

This study is limited in a few ways. First, the outcomes were self-reported. There is a need to conduct future studies to replicate these findings using administrative data. Second, the only SES indicator in this study was parental education. Parental income, wealth, and family status are other SES indicators that need to be investigated. Furthermore, we only explored the association between parental education and GPA without studying why this association varies across ethnic groups. In addition, GPA was self-reported in this study. Some research could apply other sources such as administrative and school data. Some research suggests that self-reported GPA is valid and reliable, however, it is not perfect. Finally, GPA is not the only indicator of school performance. School performance includes multiple aspects of academic success that depend on behavioral, cognitive, and emotional characteristics. Despite these limitations, this is the first study to rule out RTM as an explanation of MDRs.

## 6. Conclusions

Compared to White and non-Hispanic youth, Black and Hispanic youth gain less school performance from an increase in their parental education. For non-Hispanics and Whites, however, a perfect stepwise increase can be seen in youth school performance as parental education improves; this stepwise pattern may not exist in Black and Hispanic youth. Given that the stepwise increase in school performance is perfect for non-Hispanic and White but not in Black and Hispanic youth, and as the anomaly in shape was not limited to the end of the distribution, RTM is ruled out. Thus, RTM should not be regarded as an explanation for MDRs. Future research may explore the contextual causes of MDRs.

## Figures and Tables

**Table 1:** Descriptive data.

	All *n* = 10,262	
	*n*	%
Ethnicity		
Non-Hispanic White	6398	62.4
Hispanic White	2335	22.7
Non-Hispanic Black	1529	14.9
Age		
<18 Years	4335	42.3
18+ Years	5913	57.7
Gender		
Female	5062	51.6
Male	4739	48.4
Parents Present in the Household		
One	3258	32.0
Two	6939	68.0
Parental Education		
Some Highschool	991	10.7
Highschool Graduate	1426	15.4
Some College	1611	17.3
College Graduate	3132	33.7
Graduate School	2128	22.9
Parental Education		
Some Highschool	991	10.7
Highschool Graduate	1426	15.4
Some College	1611	17.3
College Graduate	3132	33.7
Graduate School	2128	22.9
	**Mean**	**SD**
School Performance	6.69	1.86

**Table 2:** Education level by ethnicity.

	Hispanic		Non-Hispanic White		Non-Hispanic Black	
	*n*	%	*n*	%	*n*	%
Parental Education						
Some Highschool	687	35.5	204	3.4	100	7.9
Highschool Graduate	422	21.8	778	12.8	226	17.8
Some College	349	18.0	986	16.2	276	21.7
College Graduate	270	14.0	2482	40.8	380	29.9
Graduate School	206	10.7	1632	26.8	290	22.8
All	1934	100.0	6082	100.0	1272	100.0

**Table 3:** Analysis of variance (ANOVA) results overall and by ethnicity.

	All		Non-Hispanic Whites		Blacks		Hispanics	
	Mean	SD	Mean	SD	Mean	SD	Mean	SD
Some Highschool	6.16	1.98	6.49	2.16	6.67	1.99	6.00	1.89
Highschool Graduate	6.30	1.91	6.43	1.98	6.16	1.84	6.13	1.81
Some College	6.58	1.87	6.77	1.86	6.22	1.80	6.32	1.89
College Graduate	6.86	1.80	6.99	1.78	6.43	1.76	6.21	1.87
Graduate School	7.34	1.58	7.49	1.50	6.84	1.76	6.85	1.66
*p* value	<0.001		<0.001		<0.001		<0.001	

**Table 4:** Post Hoc test overall and by ethnicity.

		All			Non-Hispanic Whites			Non-Hispanic Blacks			Hispanics		
	(J) Parental Education	Mean Difference (I-J)	SE	*p*	Mean Difference (I-J)	SE	*p*	Mean Difference (I-J)	SE	*p*	Mean Difference (I-J)	SE	*p*
**Some Highschool**	**Highschool Graduate**	−0.13	0.07	0.368	0.05	0.14	0.995	0.51	0.22	0.127	−0.13	0.11	0.764
	**Some College**	−0.42 *	0.07	<0.001	−0.29	0.14	0.215	0.45	0.21	0.206	−0.33	0.12	0.054
	**College Graduate**	−0.69 *	0.07	<0.001	−0.51 *	0.13	0.001	0.24	0.20	0.772	−0.22	0.13	0.466
	**Graduate School**	−1.18 *	0.07	<0.001	−1.01 *	0.13	<0.001	−0.17	0.21	0.924	−0.86 *	0.15	<0.001
**Highschool Graduate**	**Some Highschool**	0.13	0.07	0.368	−0.05	0.14	0.995	−0.51	0.22	0.127	0.13	0.11	0.764
	**Some College**	−0.28 *	0.07	<0.001	−0.34 *	0.08	0.001	−0.06	0.16	0.995	−0.19	0.13	0.598
	**College Graduate**	−0.56 *	0.06	<0.001	−0.56 *	0.07	<0.001	−0.27	0.15	0.365	−0.08	0.14	0.977
	**Graduate School**	−1.04 *	0.06	<0.001	−1.06 *	0.08	<0.001	−0.68 *	0.16	<0.001	−0.72 *	0.16	<0.001
**Some College**	**Some Highschool**	0.42 *	0.07	<0.001	0.29	0.14	0.215	−0.45	0.21	0.206	0.33	0.12	0.054
	**Highschool Graduate**	0.28 *	0.07	<0.001	0.34 *	0.08	0.001	0.06	0.16	0.995	0.19	0.13	0.598
	**College Graduate**	−0.28 *	0.06	<0.001	−0.22 *	0.07	0.009	−0.21	0.14	0.565	0.11	0.15	0.950
	**Graduate School**	−0.76 *	0.06	<0.001	−0.72 *	0.07	<0.001	−0.62 *	0.15	0.000	−0.53 *	0.16	0.010
**College Graduate**	**Some Highschool**	0.69 *	0.07	<0.001	0.51 *	0.13	0.001	−0.24	0.20	0.772	0.22	0.13	0.466
	**Highschool Graduate**	0.56 *	0.06	<0.001	0.56 *	0.07	<0.001	0.27	0.15	0.365	0.08	0.14	0.977
	**Some College**	0.28 *	0.06	<0.001	0.22 *	0.07	0.009	0.21	0.14	0.565	−0.11	0.15	0.950
	**Graduate School**	−0.49 *	0.05	<0.001	−0.50 *	0.06	<0.001	−0.41 *	0.14	0.031	−0.64 *	0.17	0.002
**Graduate School**	**Some Highschool**	1.18 *	0.07	<0.001	1.01 *	0.13	<0.001	0.17	0.21	0.924	0.86 *	0.15	<0.001
	**Highschool Graduate**	1.04 *	0.06	<0.001	1.06 *	0.08	<0.001	0.68 *	0.16	<0.001	0.72 *	0.16	<0.001
	**Some College**	0.76 *	0.06	<0.001	0.72 *	0.07	<0.001	0.62 *	0.15	<0.001	0.53 *	0.16	0.010
	**College Graduate**	0.49 *	0.05	<0.001	0.50 *	0.06	<0.001	0.41 *	0.14	0.031	0.64 *	0.17	0.002

* *p* < 0.05; Standard Error (SE); I and J; education levels.

**Table 5:** Post Hoc test overall and by ethnicity.

	1	2	3	4	5
**All**	n	Mean	Mean	Mean	Mean
Some Highschool	991	6.16			
Highschool Graduate	1426	6.30			
Some College	1611		6.58		
College Graduate	3132			6.86	
Graduate School	2128				7.34
***p* value**		0.212	1.000	1.000	1.000
**NHWs**					
Some Highschool	778	6.43			
Highschool Graduate	204	6.49			
Some College	986		6.77		
College Graduate	2482		6.99		
Graduate School	1632			7.49	
***p* value**		0.985	0.192	1.000	
**NHBs**					
Some Highschool	226	6.16			
Highschool Graduate	276	6.22	6.22		
Some College	380	6.43	6.43	6.43	
College Graduate	100		6.67	6.67	
Graduate School	290			6.84	
***p* value**		0.528	0.083	0.145	
**HWs**					
Some Highschool	687	6.00			
Highschool Graduate	422	6.13			
Some College	270	6.21			
College Graduate	349	6.32			
Graduate School	206		6.85		
***p* value**		0.155	1.000		

NHWs: Non-Hispanci Whites; HWs: Hiapanic Whites, NHBs: Non-Hispanic Blacks.
